# Supplementation of Vitamin D_3_ and Fructooligosaccharides Downregulates Intestinal Defensins and Reduces the Species Abundance of *Romboutsia ilealis* in C57BL/6J Mice

**DOI:** 10.3390/nu16142236

**Published:** 2024-07-11

**Authors:** Tyler Hanson, Ethan Constantine, Zack Nobles, Emily Butler, Karisa M. Renteria, Chin May Teoh, Gar Yee Koh

**Affiliations:** 1 Nutrition and Foods Program, School of Family and Consumer Sciences, Texas State University, San Marcos, TX 78666, USA; tylerhanson.univ@gmail.com (T.H.); j_n292@txstate.edu (Z.N.); ekb60@txstate.edu (E.B.); kmrenteria03@gmail.com (K.M.R.); tchinmay3@outlook.com (C.M.T.); 2 Department of Biology, College of Science and Engineering, Texas State University, San Marcos, TX 78666, USA; econstantine@txstate.edu

**Keywords:** vitamin D, fructooligosaccharides, *Romboutsia ilealis*, *Dubosiella newyorkensis*, *Akkermansia muciniphila*, beta-defensins, antimicrobial peptides

## Abstract

The activation of the vitamin D receptor (VDR) in the ileum has been shown to regulate Paneth cell-specific defensins, a large family of antimicrobial peptides; hence, this may serve as a potential mechanism to maintain intestinal homeostasis. Previously, we have demonstrated that a combination of vitamin D_3_ (VD) and fructooligosaccharides (FOSs) upregulates colonic *Vdr* in mice. Here, we aim to examine the effect of VD, alone or in combination with FOSs, on intestinal barrier integrity and the secretion of antimicrobial peptides, as well as the gut microbial community. Male and female C57BL/6J mice at 6 weeks old were randomized into three groups to receive the following dietary regimens (n = 10/sex/group) for 8 weeks: (1) standard AIN-93G control diet (CTR), (2) CTR + 5000 IU vitamin D_3_ (VD), and (3) VD + 5% fructooligosaccharides (VF). VD and VF differentially regulated the mRNA expressions of tight junction proteins in the colon and ileum. VF suppressed the upregulation of colonic *ZO-1* and *occludin*, which was induced by VD supplementation alone. In the ileum, *occludin* but not *ZO-1* was upregulated 20-fold in the VF-treated mice. While VD did not alter the mRNA expressions of *Vdr* and defensins in the ileum, these targets were downregulated by VF. Microbial analysis further reveals a shift of microbial beta diversity and a reduction in *Romboutsia ilealis*, a pathobiont, in VF-treated mice. Though the implications of these phenotypical and microbial changes remain to be determined, the administration of FOSs in the presence of VD may serve as an effective dietary intervention for maintaining intestinal homeostasis.

## 1. Introduction

Vitamin D (VD), a crucial secosteroid hormone responsible for maintaining the homeostasis of calcium, magnesium, and phosphorus mineral balance, has been documented in the literature to contribute to immune function, autoimmunity, and cancer cell apoptosis [1]. Recent studies have shown that VD and vitamin D receptor (VDR) induce positive effects on overall gut health and functioning [2,3,4]. VDR signaling and its role in the genetic modification of antimicrobial peptides (AMPs) has been explored by numerous studies [3,5,6,7,8,9]. AMPs are a class of small peptides that represent an important function of human innate immunity and consist of two main subtypes in humans, which include defensins and cathelicidins [10]. Paneth cells in the epithelial layer of the small intestine are responsible for the secretion of alpha- and beta-defensins in response to increased concentrations of pathogenic colonizing bacteria to maintain microbiome equilibrium [10,11,12]. β-defensins are targets for VDR. An *in vitro* study demonstrated that the exposure of epithelial cells to 1,25(OH)_2_D_3_, the active form of VD, promoted the expression of β-defensin 2 and the VD response element in β-defensin 2 promoter regions [12]. *In vivo*, the utilization of a VD deficiency and knockout VDR murine model led to the discovery that the combination of vitamin D deficiency and high-fat diet environment downregulated α-defensin 5 and MMP7, a known enzyme that activates pro-defensins and tight junction genes [5]. When these mice were administered α-defensin 5, the abundance of *Helicobacter hepaticus*, a pathogenic bacterium, was suppressed, while *Akkermansia muciniphila*, a reported beneficial bacterium, was enriched in the gut of these mice [5]. Others have demonstrated that *H. pylori*-infected VDR knockout mice were more susceptible to colonization and had increased VDR and cathelicidin signaling [13]. In turn, the administration of VD in *H. pylori*-infected mice resulted in the upregulation of VDR and cathelicidins [13], suggesting that the transcriptional actions of VDR on AMPs could serve as a potential mechanism for its apparent effects on the host microbiome.

The gut microbiome (GM) has been a particular topic of interest in contemporary research due to its implications for health and disease. Pathogenic bacterial species have been associated with systemic, low-grade inflammation and metabolic dysfunction that contribute to insulin resistance, hepatic lipid accumulation, cardiovascular disturbances, and immune-mediating disorders [14,15,16,17]. A balance of beneficial host bacteria and colonizing pathogenic bacteria is crucial for gut health. A VD supplementation model demonstrated improvements in GM diversity with increasing VD concentration. Interventions of VD have been shown to increase the abundance of Bacteriodes and Parabacteriodes, whereas Porphyromonas, an opportunistic pathogenic genus, was associated with lower VD status [2]. Other human clinical studies with VD supplementation show significant influences on GM diversity with improvements in the *Bacteroides*/*Firmicutes* ratio, increases in *Akkermansia* and *Bifidobacterium* taxa, and enrichment with *Prevotella* with simultaneous reductions in *Haemophilus* and *Veillonella* [17,18]. Moreover, VDR and its transcriptional actions are most likely the reason for the gut microbiome improvements seen in supplementation models. A lack of VDR in lab mice resulted in the depletion of *Lactobacillus*, *Alistipes,* and *Odoribacter* and an enrichment of *Clostridium*, *Bacteroides*, and *Eggerthella* [4]. VDR has been found to directly modulate the expression of tight junction proteins [3,5,19,20,21]. Mice lacking VDR have demonstrated increased mucosal damage from DSS-induced colitis, decreased expression of claudin, occludin, and zonula occludens-1 (ZO-1) tight junction proteins, decreased short-chain fatty acid production, and increased expression of colonic mucins [19,21,22]. Tight junction spaces between intestinal epithelial cells are responsible for preventing the translocation of endotoxins into circulation, and tight junction proteins help maintain gut barrier integrity [15]. The infiltration of pathogenic bacteria can result in endotoxemia and downstream systemic inflammation. Therefore, maintaining the integrity of the intestinal barrier and the equilibrium of gut microbial species in the gut is of particular interest for interventional studies.

Prebiotic dietary fibers are linear chains of sugar molecules that pass through the GI tract undigested until they are fermented by colonic microbiota to produce beneficial bacterial metabolites, such as short-chain fatty acids. Short-chain fatty acids, such as lactate, acetate, and pyruvate, as well as other bacterial metabolites, may have an effect on provitamin D3 synthesis [23]. Fructooligosaccharides (FOSs) consist of linear chains of fructose molecules (ranging from 2–60 units) linked by β-(2,1) glycosidic bonds [24]. FOS supplementation models in animal studies have yielded improvements in intestinal epithelial barrier function for methionine-choline-deficient mice and the amelioration of normal gastrointestinal microflora in an obesogenic model [25,26]. For their impact on colonic microflora concentration and barrier integrity, FOSs were chosen as the soluble fiber component of this intervention study.

It is evident that most of the current research has investigated the role of VD status and VDR signaling on intestinal barrier function, bacterial diversity, and the induction of AMPs. Yet, current research is lacking on the effects of VD and FOS intervention on gut health, and it is unknown whether a combination of FOSs and VD would elucidate a beneficial effect in healthy mice. Therefore, in this study, we aimed to investigate the effects of a combination of VD and FOS intervention in healthy C57BL/6J mice on gut microbiota, gene expressions of tight junction proteins, and antimicrobial peptides in the small intestine.

## 2. Materials and Methods

### 2.1. Study Design

This experiment was approved by the Institutional Animal Care and Use Committee (IACUC) at Texas State University (San Marcos, TX, USA) with an assigned protocol #8007. Upon arrival, four-week-old male and female C57BL/6J mice (The Jackson Laboratory, Bar Harbor, ME, USA) were acclimated for two weeks prior to diet assignments. During the acclimation period, all mice were fed an AIN-93G diet (Research Diets, Inc., New Brunswick, NJ, USA). At 6 weeks of age, mice were randomized (n = 10 per group per sex) to receive the following diet, respectively, for 8 weeks: standard AIN-93G diet (CTR), CTR diet supplemented with 4000 IU of VD3 (VD), VD diet supplemented with 5% *w*/*w* fructooligosaccharides (FOSs; VF). Diet was customized by Research Diets, Inc. FOSs were supplied by BENEO (Orafti-P95; Mannheim, Germany), and VD3 was supplied by Research Diets, Inc. The base diet contained 1000 IU of VD3, and hence, the addition of 4000 IU of VD3 in both the VD and VF groups yielded a final VD concentration of 5000 IU. All diets were irradiated by the vendor and were stored at −20 °C. All mice were housed individually in the specific pathogen-free facility with a 12 h light/dark cycle at 22–25 °C with ~60% humidity. Animals had unlimited access to food and water throughout the experimental period. Food was replaced with fresh pellets on a weekly basis. Body weight and food intake were recorded weekly and previously reported [27]. After 8 weeks of dietary intervention, mice were fasted overnight and anesthetized with 3% isoflurane, followed by cervical dislocation. The ileum of the small intestine and the colonic mucosa were scraped from tissues and kept at −80 °C until analysis. Cecal contents were collected at euthanasia for microbiome analysis.

### 2.2. Real-Time PCR

RNA was extracted from the mucosa cells that were collected from the colon after dissection using Trizol^®^ reagent (Invitrogen, Waltham, MA, USA). RNA was then converted to double-stranded cDNA using a SuperScript IV first-strand cDNA synthesis kit (Invitrogen, Waltham, MA, USA). SYBR Green reagent (Applied Biosystems, Waltham, MA, USA) was used for real-time PCR reactions to detect *Vdr*, tight junction proteins *ZO-1* and *occludin*, and antimicrobial peptides, alpha-defensin 1 (*Dfa1*), alpha-defensin 5 (*Dfa5*), and beta-defensin 1 (*Dfb1*). The expression of the target genes was normalized against *Gapdh*, and all data presented were expressed as a relative fold change in gene expression in CTR mice. Primers used in this study are listed in Table 1.

### 2.3. DNA Stool Extraction and 16s rRNA Sequencing

All reagents used for PCR sequencing were purchased from Thermo Fisher Scientific (Waltham, MA, USA) and Applied Biosystems, except as noted. PCR sequencing was performed using the 2-step PCR sequencing method with the Illumina MiSeq platform system. Primers for the first step of PCR sequencing included 515F (GTG CCA GCM GCC GCG GTA A) and 806R (GGA CTA CHV GGG TWT CTA AT) primers to target the V4 region. After all samples were run for the first step of PCR sequencing, the second step of PCR sequencing was started using the 16s Mi seq protocol. Both steps of the PCR sequencing were performed using the ProFlex PCR system machine™ from Applied Biosystems and were completed in triplicate. Agarose gel was used to validate the PCR products, followed by cleanup with ExoSap-IT™ (Thermo Fisher, Waltham, MA, USA) with the ProFlex PCR system. All samples had appropriate readings ranging from 15 ng/µL to 80 ng/µL using broad-range standards. The R statistical software version 4.3.2 was used to process the raw output data from the Illumina MiSeq platform. Quality control for the MiSeq readings was completed using the FAST QC package in R to remove low-quality readings. Read trimming and merging were performed with the DADA 2 package in R with the SILVA taxonomic database to cluster the gene sequence reads into OTU at 97% similarity. Microbiome Analyst was used to create figures and perform statistical analysis of the data. Data parameters on Microbiome Analyst were kept to default, excluding the low count filter, which was reduced to 15%. The read counts were normalized using total sum scaling.

### 2.4. 16s rDNA PCR Analysis

To further validate the relative abundance of target bacteria, we analyzed the expression of 16s rDNA from *Akkermansia muciniphila*, *Dubosiella newyorkensis*, and *Romboutsia ilealis* using the stool DNA (as described above in Section 2.3) of each sample. Real-time PCR was conducted utilizing the SYBR green reagent (Invitrogen, Waltham, MA, USA), and the relative abundance of the bacteria was normalized to Eubacteria expression. The bacteria primers used in this study are listed in Table 1.

### 2.5. Statistical Analysis

Statistical analyses of results from real-time PCR were performed using SigmaPlot version 14.5 (Inpixon, Palo Alto, CA, USA). The 16s rRNA PCR sequencing data were analyzed using the R statistical software, as indicated. Comparison of differences among groups was conducted using a one-way ANOVA, followed by Tukey’s multiple comparison. The mRNA expressions were determined using the delta Ct values and expressed relative to the CTR group in the figures. Statistical significance was set at *p* < 0.05, and all data were expressed as mean ± SEM unless otherwise specified.

## 3. Results

### 3.1. VD and VF Differentially Regulate the mRNA Expressions of Tight Junction Proteins in the Colon and Ileum

**Figure 1:**
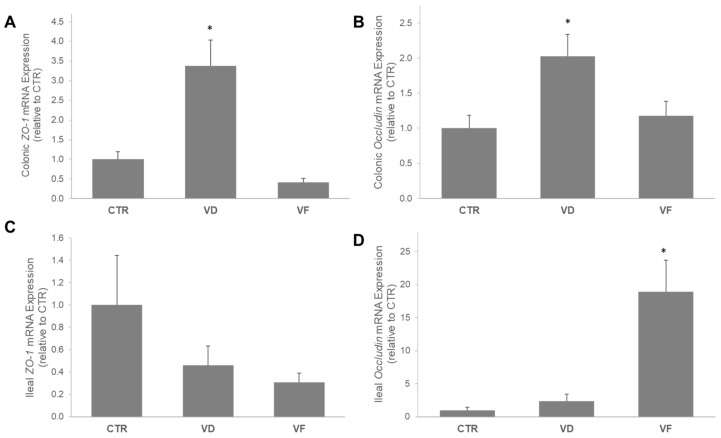
The combination of VD and FOSs differentially affects the mRNA expressions of *ZO-1* and *occludin* in the colon (**A**,**B**) and ileum (**C**,**D**). Statistical differences between dietary interventions are expressed as * *p* < 0.05. Data are expressed as mean ± standard error (n = 5–6/group/sex). CTR, control; VD, vitamin D_3_; VF, vitamin D_3_ + fructooligosaccharides.

No sex differences were observed; therefore, all data were combined for statistical analysis. Intestinal barrier integrity was assessed by measuring the mRNA expression of tight junction proteins *ZO-1* and *occludin* in both the colon and the ileum. In the colon, the mRNA expression of *ZO-1* and *occludin* were 3.5- and 2-fold higher in the VD-supplemented mice than in the CTR mice, respectively (Figure 1A,B). Interestingly, FOSs suppressed the elevation of colonic mRNA expressions of both TJPs that were induced by VD, and they did not differ from the CTR mice (Figure 1A,B). In contrast, mRNA expression of *ZO-1* in the ileum was not affected by the dietary intervention; yet, the addition of FOSs to VD (i.e., VF) enhanced the mRNA expression of intestinal *occludin* by 20-fold compared to the CTR mice (Figure 1C,D).

### 3.2. Combination of VD and FOSs Suppressed Vitamin D Signaling and Production of Defensins in the Ileum

**Figure 2:**
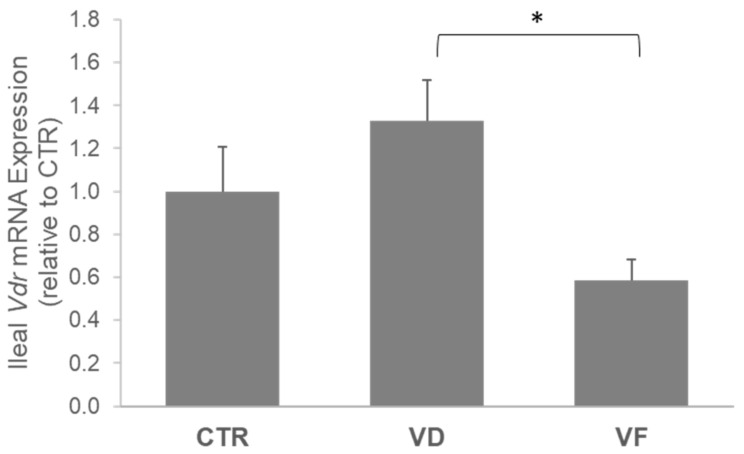
mRNA expressions of ileal *VDR*. Statistical differences between dietary interventions are expressed as * *p* < 0.05. Data are expressed as mean ± standard error (n = 6/group/sex). CTR, control; VD, vitamin D_3_; VF, vitamin D_3_ + fructooligosaccharides.

**Figure 3:**
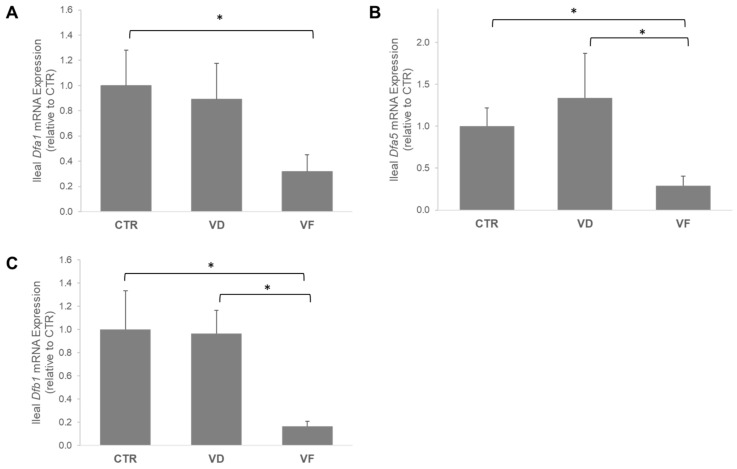
mRNA expressions of antimicrobial peptides in the ileum. (**A**) mRNA expression of *Dfa1*, (**B**) mRNA expression of *Dfa5*, and (**C**) mRNA expression of *Dfb1*. Statistical differences between dietary interventions are expressed as * *p* < 0.05. Data are expressed as mean ± standard error (n = 6/group/sex). CTR, control; VD, vitamin D_3_; VF, vitamin D_3_ + fructooligosaccharides.

VD activation in the ileum, as indicated by the mRNA expression of *VDR*, was 54% lower in VF-treated mice compared to VD mice (*p* = 0.01) and 42% lower when compared to CTR mice (*p* = 0.07) (Figure 2). Aligned with our observation in the *VDR*, the mRNA expressions of *Dfa5*, *Dfa1*, and *Dfb1* were downregulated by VF treatment compared to CTR and VD, respectively (Figure 3). We further demonstrated a positive correlation between the mRNA expression of *VDR* and mRNA expressions of *Dfa1* (Pearson’s coefficient = 0.561; *p* < 0.001) and *Dfa5* (Pearson’s coefficient = 0.519; *p* = 0.002) but not *Dfb1* (Table 2), suggesting an important role of VDR in regulating the secretions of alpha-defensins in the ileum.

### 3.3. Combination of VD and FOS Shifted the Gut Microbial Diversity and Altered Selected Gut Bacteria Species in Mice

**Figure 4:**
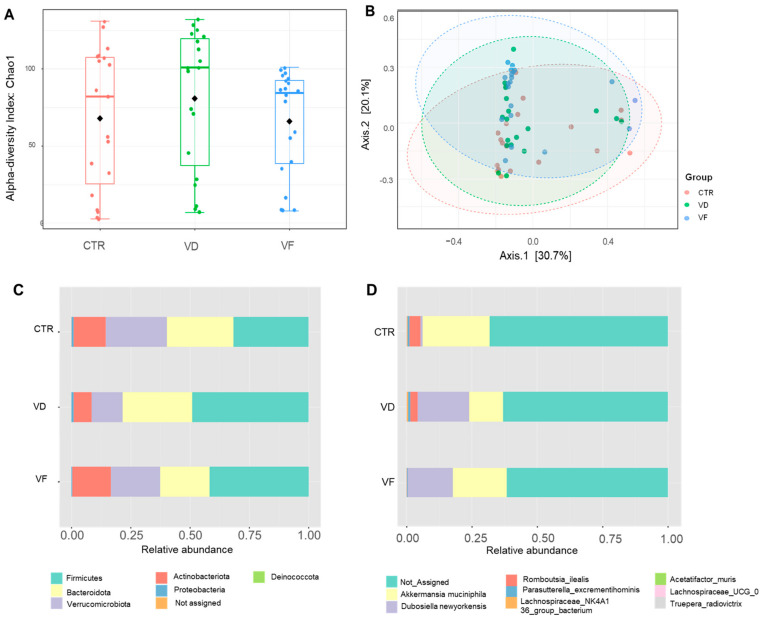
The effect of VD, alone or in combination with FOS, on cecal microbiota diversity and composition in mice. (**A**) Alpha diversity Chao 1 index, (**B**) principal component plot using bray distance, (**C**) relative abundance of gut microbiota at phylum level, and (**D**) relative abundance of gut microbiota at species level. CTR 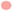, control; VD 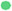, vitamin D_3_; VF 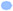, vitamin D_3_ + fructooligosaccharides. ◆, Mean of Chao 1 index.

While alpha diversity, as measured by the Chao1 index, was not significantly different among all dietary groups, the beta diversity of VF-treated mice was significantly different from both CTR (*p* < 0.001) and VD (*p* < 0.001) mice, respectively (Figure 4). No difference in beta diversity was reported between CTR and VD mice (*p* = 0.13).

**Figure 5:**
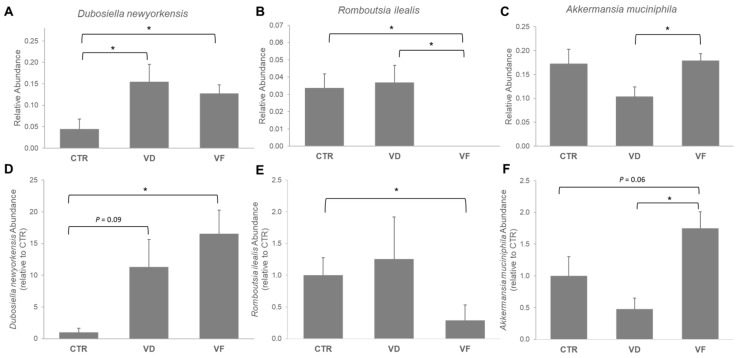
Effect of VD, alone or in combination with FOSs, on the abundance of (**A**,**D**) *Dubosiella newyorkensis*, (**B**,**E**) *Romboutsia ilealis*, and (**C**,**F**) *Akkermansia muciniphila* cecal microbiota composition in mice analyzed by 16s rRNA sequencing (**A**–**C**) and RT-PCR (**D**–**F**). Statistical differences between dietary interventions are expressed as * *p* < 0.05. Data are expressed as mean ± standard error (n = 6/group/sex). CTR, control; VD, vitamin D_3_; VF, vitamin D_3_ + fructooligosaccharides.

Our 16s rRNA analysis revealed that the average composition of the gut microbiome consists mostly of the phyla *Firmicutes* and *Bacteroidota*. Specifically, *Firmicutes* had the most abundance of all treatment groups. *Bacteroidota* had the second most available abundance of all treatment groups, followed by *Verrucomicrobiota*. The abundance of the major phyla detected in the cecal content of each dietary group is shown in Figure 4. The CTR group had a more evenly distributed gut microbiome, with nearly equal relative abundance of the phyla *Firmicutes*, *Bacteroidota*, and *Verrucomicrobiota*. Administration of either VD or VF did not alter the gut microbiota in mice at the phylum level when compared to CTR. However, the relative abundance of Proteobacteria and *Verrucomicrobiota* in VF-treated mice was different from VD-treated mice, suggesting a possible role of FOSs in restoring the changes in gut microbiota composition induced by VD administration. At the genus level, *Lactococcus*, *Akkermansia*, *Bacteroides*, and *Dubosiella* accounted for approximately 50% of the gut microbiome composition for all groups (Figure S1). Interestingly, species classification of the gut microbiome revealed that VD and VF differentially modulated *Dubosiella newyorkensis* and *Romboutsia ilealis*, respectively (Figure 5A,B). Specifically, both VD and VF enhanced the relative abundance of *D. newyorkensis*, while the relative abundance of *R. ilealis* was suppressed by VF but not with VD when compared to CTR mice (Figure 5A,B). The relative abundance of these bacteria species was further confirmed using RT-PCR (Figure 5D,E). While our 16s rRNA data demonstrated a decreased abundance of *A. muciniphila* in VD mice compared to VF-treated mice (Figure 5C), RT-PCR results further revealed an increasing trend with *A. muciniphila* in VF-treated mice (*p* = 0.06) compared to CTR and greater abundance compared to VD-treated mice (Figure 5F).

## 4. Discussion

The present study was aimed at investigating the potential role of VD supplementation in combination with FOSs on the host GM and overall indicators of gut health in healthy C57BL/6J mice. Here, we demonstrated that VD and VF differentially affect intestinal barrier integrity and the abundance of select bacteria species. Specifically, while VD enhances the mRNA expressions of ZO-1 and occludin in the colon, the combination of VD and FOSs (i.e., VF) suppresses the elevations of these tight junction proteins induced by VD. A different observation was reported in the ileum, where no changes were noted with ZO-1, but VF greatly enhanced the mRNA expression of occludin in the mice compared to CTR. In contrast with the ileal occludin in VF-treated mice, we observed an attenuation of VD signaling and mRNA expression of AMPs in the ileum. It is well documented in the literature that VDR and AMP expressions are positively correlated, and VDR directly modulates the transcriptional expression of AMPs in the small intestine through Paneth-cell excretions [3,5,6,7,8,9,20]. The positive correlation between VDR expression and defensin expression found in the literature matched the results of the present study. Interestingly, while CTR and VD mice did not differ, the VF mice showed significantly reduced gene expression of *Vdr* and all small intestine defensins analyzed. The exact reason for this correlation is not entirely elucidated, and there may be a few possible physiological explanations for this occurrence.

First, FOS supplementation in the VF group may hinder the absorption of vitamin D in the intestinal lumen. This could occur because FOSs are soluble fibers that can create a gelatinous-like substance in the intestinal environment that may hinder micelle formation and increase the viscosity of chyme, resulting in impaired VD diffusion into enterocytes [30]. Perhaps the accumulation of fluid to create fibrous gel formulation in the small intestine prevented VD absorption. It was also observed that serum 1,25(OH)_2_D_3_ concentration was elevated in the VF-fed mice compared to CTR and VD, as previously described [27]. Hence, we speculated that the potential blockage of VD absorption in the intestinal lumen may have created a feedback mechanism associated with the physiological need for 1,25(OH)_2_D_3_, causing an increased renal production of calcitriol through 1-alpha-hydroxylase activity. Another possible speculation for diminished *Vdr* expression in the VF group could be due to the presence of a compensatory mechanism, in which the increased VDR concentration could decrease the need for transcription of *Vdr* in the VF group. It is demonstrated through VDR knockout models that AMP secretion is exponentially increased [5,13,31]. Perhaps an overabundance of VDR protein concentration in the VF group produced the opposite effect of VDR KO models and signaled the inhibition of AMP transcriptional expression. This claim can be alluded to from the present study, but this mechanism would have to be explored further to confirm by assessing the protein expression of VDR in the intestinal lumen. It should be noted that we have previously shown that the colonic *Vdr* mRNA expression was induced by VF [27], an observation that is inversely correlated with the ileum. We hypothesized that the elevation in the colon could be a result of FOS-mediated changes in the GM and microbial metabolites that may directly induce VDR activity. Further *in vitro* studies have displayed the direct expression of VDR by microbial metabolites, such as SCFAs, that can stimulate the induction of toll-like receptor pathways [32,33,34], suggesting that the differential VDR stimulation along the gastrointestinal tract could be dependent on exogenous factors like commensal bacteria.

Tight junction proteins are crucial for the normal integrity of paracellular spaces between intestinal mucosa cells. The expression of the tight junction proteins occludin and ZO-1 of the small intestine and colon were analyzed in this study. In the small intestine, no changes were observed between CTR and VD mice. Interestingly, the VF group enhanced the mRNA expression of *occludin* by 20-fold when compared to CTR and VD, respectively. FOSs have been shown to promote intestinal barrier integrity [35,36,37]. Thus, we hypothesize that the improved intestinal homeostasis by FOSs may lead to less physiological desire for AMPs and its key regulator, VDR, which may partially explain the increased *occludin* and decreased *VDR* and AMP expressions in the ileum. It should be noted that occludin does not represent the entirety of the overall tight junction integrity, but it does indicate an initial positive outcome in terms of intestinal barrier integrity. In the present study, the colonic TJP results contradicted the results from the ileum. The expressions of colonic *ZO-1* and *occludin* were significantly lower in the VF group compared to VD. The exact reasoning behind these variations in results is not fully understood. FOSs may play a role in the colonic cellular environment in regard to the regulation of TJP expression. The combined effects of VD and FOSs on the microbiome may have saturated the colonic lumen with beneficial microbial metabolites that electively downregulate the expression of new TJP due to decreased necessity. This effect is not observed in the VD group, perhaps because the lack of FOS creates a less proliferative microbial environment and decreases microbial metabolism. The elevated TJP expression in the VD group is characteristic of evidence from the literature, signifying that VDR activity has a direct effect on the expression of TJP, specifically in the colon [3,5,19,22]. Taken together, the varied outcomes observed in the colon and small intestine may indicate a close interaction between VD signaling, intestinal permeability, and the gastrointestinal microenvironment.

The present study demonstrated that the administration of VD, with or without the presence of FOSs, resulted in an increased relative abundance of *Dubosiella newyorkensis* and a decreased relative abundance of *Romboutsia ilealis* from fecal samples analyzed through 16S sequencing data. While neither VD- nor VF-treated mice significantly impacted the relative abundance of *Akkermansia muciniphila*, an increasing trend was observed in VF mice when compared to CTR mice, which was further confirmed with RT-PCR analysis. *A. muciniphila* and *D. newyorkensis* were the two most abundant species found through taxonomic composition analysis of the sample bacterial community. Evidence suggests that these two bacterial species are beneficial in the gut [25,38,39,40]. *A. muciniphila* is a known gut flora species that has shown increased concentration in healthy individuals along the lifespan, strong SCFA-producing capabilities, and overall modulation of intestinal and metabolic health [41]. *D. newyorkensis* is a novel, less extensively studied species but has been found to have beneficial properties in dextran sulfate sodium-induced colitis rat models [13] and increased abundance following fibrous berry powder supplementation in obese mice [39]. *Romboutsia ilealis*, in contrast, is known as a pathobiont and is often associated with the consumption of a Western diet and metabolic disorders, such as obesity and diabetes [42,43,44]. Consistent with the shift in potentially beneficial bacteria in our study, our results demonstrated a decreased abundance of *R. ilealis* in VF mice when compared to CTR, despite no changes being observed in the VD group. Therefore, an abundance of these selected bacterial species may indicate an effect of FOS intervention on the overall diversity of gut flora in our study.

It was observed that mice in the VF group displayed significant differences in the beta diversity of the GM compared to those in the VD and CTR groups, respectively. At the phylum level, *Firmicutes*, the phylum to which *D. newyorkensis* and *R. ilealis* belong, remained the most abundant among all treatment groups. An increased relative abundance of *Verrucomicrobiota* was observed in the VF- and CTR-treated mice, which is the phylum from which the beneficial *A. muciniphila* stems. This shift in the bacteria composition at the phylum level could indicate an overall balancing effect on the GM from VF intervention. Yet, there were no observed significant differences in alpha diversity among the groups, which may be attributed to the fact that all mice were otherwise healthy despite the supplementation of VD and FOSs. Perhaps we could observe more stark differences in bacterial abundance if the mice model was a disease model or an obesogenic study design. These data on changes to the beta-diversity and abundance of *Romboutsia ilealis*, *A. muciniphila,* and *D. newyorkensis* at the species and phylum level indicate proof-of-concept correlations between VD and FOS supplementation and improvements to the overall GM composition. Further studies would need to be conducted to confirm the health implications of the GM composition changes observed in this study.

As mentioned previously, VD signaling activity through VDR has been associated with improved overall GM profiles and metabolic factors, such as the induction of AMP-mediated immune responses. There is an observed correlation between all AMPs with mRNA expression in the VF group, showing significantly decreased AMP expression in the small intestine compared to the VD and CTR groups. This decreased expression could be a result of improved GM composition from FOS supplementation in the VF group. Colonic fermentation of FOSs may have created a more balanced microflora composition throughout the gut, which could have created a negative feedback mechanism for AMP secretion. With natural improvements in the GM caused by FOSs, there could be less of a need for the AMP-mediated modulation of gut flora. This correlation between decreased AMP secretion and improvements in the GM may be crucial for maintaining intestinal homeostasis. Though it is beyond the scope of our investigation, the impact of the GM on gut-associated lymphoid tissue may also serve as a critical mechanism that could indirectly alter the mucosa immune system, including the secretion of AMPs. Further investigations are warranted to elucidate the interactions between the GM and the gut-associated lymphoid system, as well as the impacts resulting from VD and FOSs.

The strengths of this study include the utilization of both male and female mice, though sex difference was not a major contributor to the measured outcomes. This is also the first study investigating the effect of FOSs, in combination with VD, on VDR signaling in the small intestine and the subsequent impact on the secretions of alpha- and beta-defensins or Paneth cell-specific antimicrobial peptides. While it is still unclear how these AMPs may contribute to GM changes, we have observed a shift in gut bacteria, with an increased abundance of beneficial bacteria, such as *A. muciniphila* and *D. newyorkensis*, and a decreased abundance of the potentially opportunistic pathogen, *R. ilealis*, upon VF administration. We speculated that there are significant interactions between AMPs and changes in these gut microbiota that could potentially contribute to maintaining human health. Some limitations associated with this study are the exclusion of the FOSs alone group and the lack of information with regard to the protein concentrations of VDR, defensins, and tight junction proteins to confirm the results of PCR. The initial hypothesis of this study is to evaluate the effect of FOSs on VD metabolism. We did not anticipate FOSs to alter the basal level of VD under healthy conditions. Hence, we did not include a group with FOSs alone when conducting this experiment. While we cannot rule out whether VD exerts synergistic or additive effects with FOSs, VF mice but not VD mice tend to promote changes in epithelial barrier integrity and production of defensins, suggesting that FOSs alone are sufficient to induce these effects. Additionally, the OTU clustering approach may be a limitation, as this approach may increase the risk of grouping similar species into a single OTU compared to taxa assignments based on ASVs. Therefore, the abundance of the selected bacteria among all groups was further validated via RT-PCR, and it was consistent with our 16s rRNA sequencing results.

## 5. Conclusions

Our data demonstrated that the combination of FOSs and VD but not VD alone downregulates VDR mRNA expression and its downstream target of the antimicrobial peptides and upregulates the gene expression of occludin, a well-documented tight junction protein, in the ileum. As FOSs have been shown to modulate gut microbiota, our results may suggest a potential interaction between the gut microbiota and VDR, a mechanism that could impact the subsequent production of defensins and tight junction proteins. Because FOSs are a prebiotic fiber, gut microbiota changes were further characterized. A shift in gut bacteria was observed with VF mice when compared to CTR or VD mice, respectively, indicating that FOSs are capable of modulating the gut microbial community. However, whether these changes are directly associated with the reported phenotypes remained unclear. Future investigations are warranted to determine the health implications of VDR activation and subsequent alpha-defensin production, as well as changes in specific gut microbiota upon the consumption of FOSs.

## Figures and Tables

**Table 1:** Primer pairs used in real-time PCR analysis.

Primer	Forward (5′–3′)	Reverse (5′–3′)
*Gapdh*	GGAGAAACCTGCCAAGTA	AAGAGTGGGAGTTGCTGTTG
*Vdr*	ATGGAGGCAATGGCAGCCAGCACCTC	GAAACCCTTGCAGCCTTCACAGGTCA
*ZO-1*	GCC GCT AAG AGC ACA GCA A	GCC CTC CTT TTA ACA CAT CAG A
*Occludin*	GTG AAT GGG TCA CCG AGG G	AGA TAA GCG AAC CTG CCG AG
*Dfa1*	GGCTCCTGCTCACCAATTCT	GCCTCAGAGCTGATGGTTGT
*Dfa5*	GCTCCTGCTCAACAATTCTCC	CAGCTGCAGCAGAATACGA
*Dfb1*	ACACCCCATCTGCAACCTTA	TGTCCAAGTCCCAACACAGA
*Akkermansia muciniphila* [5]	CAGCACGTGAAGGTGGGGAC	CCTTGCGGTTGGCTTCAGAT
*Dubosiella newyorkensis*	CGAGGAAGGTCTTCGGATCG	AGGACTCACTGCGTTGACTG
*Romboutsia ilealis* [28]	GGGGCTAGCGTTATTCCGAA	CACCTGTCACTTCTGTCCCC
Eubacteria (Universal) [29]	ACT CCT ACG GGA GGC AGC AGT	ATT ACC GCG GCT GCT GGC

**Table 2:** Pearson’s correlations between mRNA expressions of alpha- and beta-defensins and *VDR* in the ileum of C57BL/6J mice.

	Ileal mRNA Expressions		
	*DFa5*	*DFa1*	*DFb1*
Pearson’s correlation coefficient (r) with ileal VDR	0.561	0.519	−0.028
*p*-value	<0.01	<0.01	0.87

## Data Availability

The original contributions presented in the study are included in the article/Supplementary Materials, further inquiries can be directed to the corresponding author.

## Supplementary Materials

The following supporting information can be downloaded at: https://www.mdpi.com/article/10.3390/nu16142236/s1, Figure S1: Relative abundance of cecal microbiota at Genus level.

## References

[1] Holick M.F. (2004). Sunlight and vitamin D for bone health and prevention of autoimmune diseases, cancers, and cardiovascular disease. Am. J. Clin. Nutr..

[2] Charoenngam N., Shirvani A., Kalajian T.A., Song A., Holick M.F. (2020). The Effect of Various Doses of Oral Vitamin D(3) Supplementation on Gut Microbiota in Healthy Adults: A Randomized, Double-blinded, Dose-response Study. Anticancer Res..

[3] Del Pinto R., Ferri C., Cominelli F. (2017). Vitamin D Axis in Inflammatory Bowel Diseases: Role, Current Uses and Future Perspectives. Int. J. Mol. Sci..

[4] Jin D., Wu S., Zhang Y.G., Lu R., Xia Y., Dong H., Sun J. (2015). Lack of Vitamin D Receptor Causes Dysbiosis and Changes the Functions of the Murine Intestinal Microbiome. Clin. Ther..

[5] Su D., Nie Y., Zhu A., Chen Z., Wu P., Zhang L., Luo M., Sun Q., Cai L., Lai Y. (2016). Vitamin D Signaling through Induction of Paneth Cell Defensins Maintains Gut Microbiota and Improves Metabolic Disorders and Hepatic Steatosis in Animal Models. Front. Physiol..

[6] Chung C., Silwal P., Kim I., Modlin R.L., Jo E.K. (2020). Vitamin D-Cathelicidin Axis: At the Crossroads between Protective Immunity and Pathological Inflammation during Infection. Immune Netw..

[7] Bishop E.L., Ismailova A., Dimeloe S., Hewison M., White J.H. (2021). Vitamin D and Immune Regulation: Antibacterial, Antiviral, Anti-Inflammatory. JBMR Plus.

[8] Dimitrov V., White J.H. (2016). Species-specific regulation of innate immunity by vitamin D signaling. J. Steroid Biochem. Mol. Biol..

[9] Guo C., Sinnott B., Niu B., Lowry M.B., Fantacone M.L., Gombart A.F. (2014). Synergistic induction of human cathelicidin antimicrobial peptide gene expression by vitamin D and stilbenoids. Mol. Nutr. Food Res..

[10] Huan Y., Kong Q., Mou H., Yi H. (2020). Antimicrobial Peptides: Classification, Design, Application and Research Progress in Multiple Fields. Front. Microbiol..

[11] Bevins C.L., Salzman N.H. (2011). Paneth cells, antimicrobial peptides and maintenance of intestinal homeostasis. Nat. Rev. Microbiol..

[12] Wang T.T., Nestel F.P., Bourdeau V., Nagai Y., Wang Q., Liao J., Tavera-Mendoza L., Lin R., Hanrahan J.W., Mader S. (2004). Cutting edge: 1,25-dihydroxyvitamin D3 is a direct inducer of antimicrobial peptide gene expression. J. Immunol..

[13] Zhang Y., Wang C., Zhang L., Yu J., Yuan W., Li L. (2022). Vitamin D(3) eradicates Helicobacter pylori by inducing VDR-CAMP signaling. Front. Microbiol..

[14] Canfora E.E., Meex R.C.R., Venema K., Blaak E.E. (2019). Gut microbial metabolites in obesity, NAFLD and T2DM. Nat. Rev. Endocrinol..

[15] Takeuchi T., Kubota T., Nakanishi Y., Tsugawa H., Suda W., Kwon A.T., Yazaki J., Ikeda K., Nemoto S., Mochizuki Y. (2023). Gut microbial carbohydrate metabolism contributes to insulin resistance. Nature.

[16] Kang Y., Kang X., Yang H., Liu H., Yang X., Liu Q., Tian H., Xue Y., Ren P., Kuang X. (2022). Lactobacillus acidophilus ameliorates obesity in mice through modulation of gut microbiota dysbiosis and intestinal permeability. Pharmacol. Res..

[17] Luthold R.V., Fernandes G.R., Franco-de-Moraes A.C., Folchetti L.G., Ferreira S.R. (2017). Gut microbiota interactions with the immunomodulatory role of vitamin D in normal individuals. Metabolism.

[18] Singh P., Rawat A., Alwakeel M., Sharif E., Al Khodor S. (2020). The potential role of vitamin D supplementation as a gut microbiota modifier in healthy individuals. Sci. Rep..

[19] Kong J., Zhang Z., Musch M.W., Ning G., Sun J., Hart J., Bissonnette M., Li Y.C. (2008). Novel role of the vitamin D receptor in maintaining the integrity of the intestinal mucosal barrier. Am. J. Physiol. Gastrointest. Liver Physiol..

[20] Sun J. (2016). VDR/vitamin D receptor regulates autophagic activity through ATG16L1. Autophagy.

[21] Zhu W., Yan J., Zhi C., Zhou Q., Yuan X. (2019). 1,25(OH)2D3 deficiency-induced gut microbial dysbiosis degrades the colonic mucus barrier in Cyp27b1 knockout mouse model. Gut Pathog..

[22] Sun J., Zhang Y.G. (2022). Vitamin D Receptor Influences Intestinal Barriers in Health and Disease. Cells.

[23] Gokhale S., Bhaduri A. (2019). Provitamin D3 modulation through prebiotics supplementation: Simulation based assessment. Sci. Rep..

[24] Sabater-Molina M., Larque E., Torrella F., Zamora S. (2009). Dietary fructooligosaccharides and potential benefits on health. J. Physiol. Biochem..

[25] Liu T.W., Cephas K.D., Holscher H.D., Kerr K.R., Mangian H.F., Tappenden K.A., Swanson K.S. (2016). Nondigestible Fructans Alter Gastrointestinal Barrier Function, Gene Expression, Histomorphology, and the Microbiota Profiles of Diet-Induced Obese C57BL/6J Mice. J. Nutr..

[26] Matsumoto K., Ichimura M., Tsuneyama K., Moritoki Y., Tsunashima H., Omagari K., Hara M., Yasuda I., Miyakawa H., Kikuchi K. (2017). Fructo-oligosaccharides and intestinal barrier function in a methionine-choline-deficient mouse model of nonalcoholic steatohepatitis. PLoS ONE.

[27] Renteria K.M., Constantine E., Teoh C.M., Cooper A., Lozano N., Bauer S., Koh G.Y. (2024). Combination of vitamin D(3) and fructooligosaccharides upregulates colonic vitamin D receptor in C57BL/6J mice and affects anxiety-related behavior in a sex-specific manner. Nutr. Res..

[28] Kim D., Yan J., Bak J., Park J., Lee H., Kim H. (2022). Sargassum thunbergii Extract Attenuates High-Fat Diet-Induced Obesity in Mice by Modulating AMPK Activation and the Gut Microbiota. Foods.

[29] Vaishnava S., Yamamoto M., Severson K.M., Ruhn K.A., Yu X., Koren O., Ley R., Wakeland E.K., Hooper L.V. (2011). The antibacterial lectin RegIIIgamma promotes the spatial segregation of microbiota and host in the intestine. Science.

[30] Maurya V.K., Aggarwal M. (2017). Factors influencing the absorption of vitamin D in GIT: An overview. J. Food Sci. Technol..

[31] Wu S., Zhang Y.G., Lu R., Xia Y., Zhou D., Petrof E.O., Claud E.C., Chen D., Chang E.B., Carmeliet G. (2015). Intestinal epithelial vitamin D receptor deletion leads to defective autophagy in colitis. Gut.

[32] Daniel C., Schroder O., Zahn N., Gaschott T., Steinhilber D., Stein J.M. (2007). The TGFbeta/Smad 3-signaling pathway is involved in butyrate-mediated vitamin D receptor (VDR)-expression. J. Cell Biochem..

[33] Moreno-Torres M., Guzmán C., Petrov P.D., Jover R. (2022). Valproate and Short-Chain Fatty Acids Activate Transcription of the Human Vitamin D Receptor Gene through a Proximal GC-Rich DNA Region Containing Two Putative Sp1 Binding Sites. Nutrients.

[34] Campbell G.R., Spector S.A. (2012). Toll-like receptor 8 ligands activate a vitamin D mediated autophagic response that inhibits human immunodeficiency virus type 1. PLoS Pathog..

[35] Wongkrasant P., Pongkorpsakol P., Ariyadamrongkwan J., Meesomboon R., Satitsri S., Pichyangkura R., Barrett K.E., Muanprasat C. (2020). A prebiotic fructo-oligosaccharide promotes tight junction assembly in intestinal epithelial cells via an AMPK-dependent pathway. Biomed. Pharmacother..

[36] Ten Bruggencate S.J., Bovee-Oudenhoven I.M., Lettink-Wissink M.L., Katan M.B., van der Meer R. (2006). Dietary fructooligosaccharides affect intestinal barrier function in healthy men. J. Nutr..

[37] Ma Q., Zhang X., Xu X., Lu Y., Chen Q., Chen Y., Liu C., Chen K. (2023). Long-term oral administration of burdock fructooligosaccharide alleviates DSS-induced colitis in mice by mediating anti-inflammatory effects and protection of intestinal barrier function. Immun. Inflamm. Dis..

[38] Geesala R., Recharla N., Zhang K., Johnson J.C., Golovko G., Khanipov K., Brining D.L., Shi X.-Z. (2024). Exclusive Enteral Nutrition Beneficially Modulates Gut Microbiome in a Preclinical Model of Crohn’s-like Colitis. Nutrients.

[39] Rodríguez-Daza M.C., Roquim M., Dudonné S., Pilon G., Levy E., Marette A., Roy D., Desjardins Y. (2020). Berry Polyphenols and Fibers Modulate Distinct Microbial Metabolic Functions and Gut Microbiota Enterotype-Like Clustering in Obese Mice. Front. Microbiol..

[40] Zhang Y., Tu S., Ji X., Wu J., Meng J., Gao J., Shao X., Shi S., Wang G., Qiu J. (2024). Dubosiella newyorkensis modulates immune tolerance in colitis via the L-lysine-activated AhR-IDO1-Kyn pathway. Nat. Commun..

[41] Chiantera V., Laganà A.S., Basciani S., Nordio M., Bizzarri M. (2023). A Critical Perspective on the Supplementation of Akkermansia muciniphila: Benefits and Harms. Life.

[42] Rodrigues R.R., Gurung M., Li Z., García-Jaramillo M., Greer R., Gaulke C., Bauchinger F., You H., Pederson J.W., Vasquez-Perez S. (2021). Transkingdom interactions between Lactobacilli and hepatic mitochondria attenuate western diet-induced diabetes. Nat. Commun..

[43] Morissette A., André D.M., Agrinier A.L., Varin T.V., Pilon G., Flamand N., Houde V.P., Marette A. (2023). The metabolic benefits of substituting sucrose for maple syrup are associated with a shift in carbohydrate digestion and gut microbiota composition in high-fat high-sucrose diet-fed mice. Am. J. Physiol. Endocrinol. Metab..

[44] Zhu C.H., Li Y.X., Xu Y.C., Wang N.N., Yan Q.J., Jiang Z.Q. (2023). Tamarind Xyloglucan Oligosaccharides Attenuate Metabolic Disorders via the Gut-Liver Axis in Mice with High-Fat-Diet-Induced Obesity. Foods.

