# Supplementary figures and images for: Supplementation of Vitamin D3 and Fructooligosaccharides Downregulates Intestinal Defensins and Reduces the Species Abundance of Romboutsia ilealis in C57BL/6J Mice
Source: Nutrients. 2024 Jul 11;16(14):2236. doi: 10.3390/nu16142236 (PMC11280458; doi:10.3390/nu16142236)

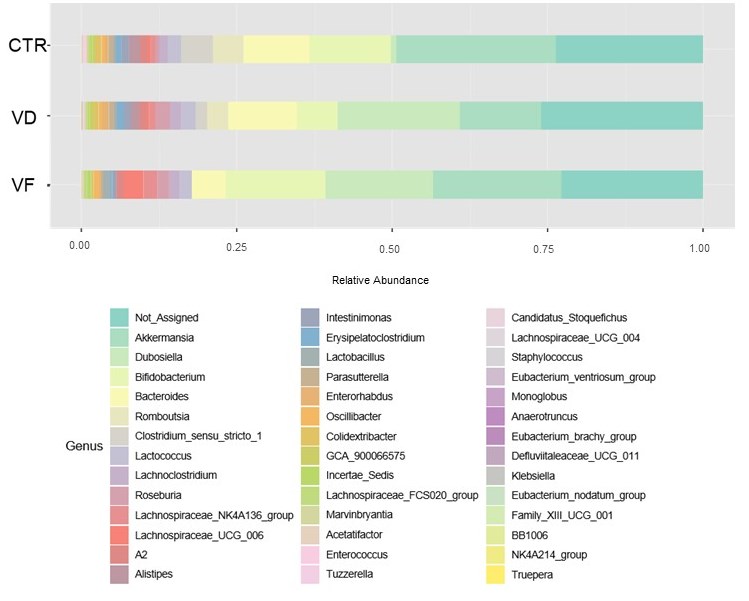

Supplement: Supplementary file 1 [file nutrients-16-02236-s001.zip › nutrients-3081651-supplementary.jpg]
